# Bacterial Cellulose Membranes Functionalized with In Situ Green-Synthesized Silver Nanoparticles for Antibacterial Applications

**DOI:** 10.3390/ijms27093943

**Published:** 2026-04-28

**Authors:** Gul Naz Ashraf, Marta Palau Gauthier, Qiutian She, Pilar Rivera-Gil, Javier Macia

**Affiliations:** 1 Synthetic Biology for Biomedical Applications Lab, Department of Medicine and Health Sciences, Universitat Pompeu Fabra, Biomedical Research Park, 08003 Barcelona, Spain; gulnaz.ashraf@upf.edu (G.N.A.); marta.palaui@upf.edu (M.P.G.); 2 Integrative Biomedical Materials and Nanomedicine Lab, Department of Medicine and Health Sciences, Universitat Pompeu Fabra, Biomedical Research Park, 08003 Barcelona, Spain; qiutian.she@upf.edu (Q.S.); pilar.rivera@upf.edu (P.R.-G.)

**Keywords:** plant-mediated nanoparticle synthesis, bactericidal composite materials, silver-based nanomaterials, biopolymer–metal nanocomposites

## Abstract

This study demonstrates how synthesis conditions and bacterial cellulose (BC) functionalization influence the physicochemical properties and antibacterial performance of BC membranes containing green-synthesized silver nanoparticles (AgNPs). Mint and avocado-seed extracts enabled AgNP formation in aqueous media but differed in composition. UV–Vis screening across pH and temperature revealed inefficient synthesis at acidic pH, whereas higher temperatures produced broader localized surface plasmon resonance (LSPR) bands. Neutral conditions generated the most intense and narrow LSPR signals. Under optimized conditions (pH 7, 23 °C), AgNPs were confirmed by TEM, and their colloidal properties differed between extracts: mint-derived particles exhibited smaller hydrodynamic diameters and lower polydispersity than avocado-derived AgNPs. Two BC functionalization strategies were evaluated: immersion in pre-formed AgNP dispersions and in situ synthesis within the BC matrix. In situ membranes displayed stronger and better-defined LSPR peaks. Agitation released nanoparticles from all BC-AgNP membranes, with smaller species released from in situ systems. Antibacterial assays against *E. coli* showed greater bactericidal activity for in situ membranes. Avocado-derived in situ BC-AgNPs produced larger inhibition halos and prevented bacterial regrowth in liquid culture. Overall, in situ green synthesis within BC provides an effective route to robust and sustainable antibacterial BC membranes.

## 1. Introduction

Nanoparticles have attracted considerable attention due to their distinctive physicochemical properties, including a high surface-to-volume ratio, tunable size and shape, and versatile surface chemistry, which enable a wide range of technological and biomedical applications [1,2,3]. Among them, silver nanoparticles (AgNPs) are particularly relevant because of their well-established antimicrobial activity and their successful integration into functional materials and membrane-based systems [4,5,6]. In healthcare, AgNPs are widely used in antimicrobial wound dressings and burn treatments and are increasingly incorporated into composite membranes such as nanofiber mats, hydrogels, and polymeric scaffolds. In these systems, nanoparticle embedding improves mechanical stability, enables controlled release, and promotes a homogeneous distribution of the active agent [7,8,9].

Conventional AgNP synthesis methods often rely on physical or chemical approaches involving hazardous reagents and the generation of toxic by-products [10]. In contrast, green synthesis strategies based on biological or plant-derived reducing and stabilizing agents operate under mild conditions, improve biocompatibility, allow control over nanoparticle nucleation and growth, and align with sustainability principles [11,12]. The integration of green-synthesized AgNPs into functional membranes, therefore, represents a promising route for the development of environmentally benign and application-ready nanocomposites.

In this context, the design of functional membranes that embed green-synthesized AgNPs becomes especially compelling. Among biopolymeric scaffolds, bacterial cellulose (BC) is an attractive biopolymeric scaffold for such applications due to its high purity, ultrafine nanofibrillar network, excellent mechanical strength, high water-holding capacity, and intrinsic biocompatibility [13]. However, conventional nanoparticle-loading methods, including post-synthesis impregnation or surface adsorption, often result in limited penetration, heterogeneous distribution, and variable loading efficiency [14,15]. In situ nanoparticle synthesis within the BC matrix overcomes these limitations by enabling the direct reduction of metal precursors (e.g., AgNO_3_) into nanoparticles embedded throughout the cellulose network, leading to improved dispersion, stronger matrix–nanoparticle interactions, and enhanced stability of the resulting nanocomposite membranes [16].

In this study, the green synthesis of AgNPs using plant extracts from mint (*Mentha spicata*) and avocado seed (*Persea americana*) as dual reducing and stabilizing agents, followed by their in situ incorporation into BC membranes, was analyzed. While plant-mediated AgNP synthesis has been extensively explored using extracts from species such as *Azadirachta indica* [17], *Curcuma longa* [18], and *Moringa oleífera* [19], comparatively few studies have investigated the use of mint or avocado extracts. Differences in the phytochemical profiles of these extracts are expected to influence reduction kinetics and nanoparticle morphology [20,21]. In addition to the effects associated with phytochemical composition, the influence of synthesis parameters, including silver precursor concentration, temperature, and pH, is systematically evaluated.

Overall, by combining the structural robustness and biocompatibility of BC with an environmentally friendly AgNP synthesis strategy, this work aims to develop multifunctional nanocomposite membranes with potential across a wide range of applications, while avoiding the use of toxic reagents during nanoparticle synthesis.

## 2. Results

### 2.1. Composition of Mint and Avocado Seed Extracts

Among plant-derived constituents, phenolic compounds, flavonoids, and carbohydrates are widely recognized as key contributors to metal ion reduction and nanoparticle stabilization in green synthesis approaches [22]. Accordingly, the total contents of these components were determined to characterize the plant extracts used in this study and assess their potential role in plant-mediated nanoparticle formation. These analyses were performed in accordance with standardized methods, as described in Section 4. The chemical compositions of the mint and avocado extracts are summarized in Table 1.

Although both extracts exhibit comparable overall phenolic content, the higher flavonoid content of the mint extract is expected to enhance the reduction rate of Ag^+^ ions, promoting faster and more synchronous nucleation events. Furthermore, differences in carbohydrate content may influence not only the nucleation rate but also the growth and stabilization of metallic nanoparticles during green synthesis. An increased carbohydrate content is likely to favor a more prolonged growth phase, leading to a higher tendency toward polydispersity while simultaneously enhancing surface stabilization through increased capping effects.

### 2.2. Synthesis, Characterization and Optimization of Green-Synthesis Conditions for Silver Nanoparticles (AgNPs)

Silver nanoparticles (AgNPs) were synthesized in aqueous phase by reducing AgNO_3_ at different concentrations using plant extracts from mint and avocado, following the procedures detailed in Section 4. The aqueous-phase reactions were first monitored by the characteristic color change of the reaction mixture (Figure S1).

We then evaluated synthesis efficiency as a function of AgNO_3_ concentration, temperature, and pH.

To investigate the influence of temperature on the green synthesis of AgNPs, three representative temperatures (4 °C, 23 °C, and 70 °C) were selected to explore kinetically limited, moderate, and thermally accelerated reaction regimes. Low temperature (4 °C) was used to slow reduction kinetics and nucleation, while room temperature (23 °C) served as a mild and environmentally relevant reference condition. Elevated temperature (70 °C) was chosen to enhance Ag^+^ reduction, increase supersaturation, and promote faster nucleation, potentially affecting particle size distribution and growth dynamics [22].

Similarly, the effect of pH was evaluated at pH 5, 7, and 11 to represent acidic, neutral, and alkaline environments. Since pH influences the ionization state and reducing capacity of phytochemicals, it directly affects reduction kinetics and the nucleation–growth balance. Acidic conditions tend to slow reduction due to the protonation of functional groups, whereas alkaline conditions enhance reducing power through the deprotonation of phenolic and hydroxyl groups, often leading to faster nucleation and improved colloidal stabilization. Neutral pH provides a balanced reference condition. Together, these parameters enable a systematic assessment of the physicochemical factors governing nanoparticle formation.

Figures S2 and S3 present the UV–Vis spectra recorded for nanoparticles synthesized using avocado and mint extracts, respectively, at different AgNO_3_ concentrations (0.01 M, 0.0075 M, 0.005 M, 0.002 M, and 0.001 M). The main spectral features and their correlation with synthesis parameters (AgNO_3_ concentration, temperature, and pH) were analyzed. As shown in Figures S2 and S3, both synthesis efficiency and the optical characteristics of the nanoparticles depend on AgNO_3_ concentration, as well as on temperature and pH.

The experimental results indicate that AgNP synthesis using either avocado or mint extracts was inefficient under acidic conditions, in some cases showing no detectable localized surface plasmon resonance (LSPR) peak, suggesting limited or absent nanoparticle formation. Synthesis efficiency increased with pH, while both efficiency and nanoparticle characteristics were further influenced by temperature. At elevated temperature (70 °C), broader LSPR peaks were consistently observed, indicating a wider particle-size distribution and/or increased aggregation.

Overall, neutral pH (pH 7) and moderate temperature (23 °C) provided the most favorable conditions, yielding higher and narrower LSPR peaks. Although higher temperatures enhanced reaction kinetics, they also led to broader LSPR peaks, whereas acidic conditions strongly suppressed nanoparticle formation. It is worth noting that differences between mint and avocado extracts’ composition were reflected in distinct UV–Vis spectral profiles, indicating extract- dependent effects on nanoparticle characteristics.

Finally, the formation of nanoparticles under conditions of pH 7 and 23 °C, using both mint and avocado seed extracts, was confirmed by transmission electron microscopy (TEM), as shown in Figure S4.

Avocado-derived AgNPs (Figure S4a) show a heterogeneous population where individual nanoparticles coexist with small clusters and occasional larger aggregates, with particles displaying predominantly rounded to slightly faceted morphologies. In contrast, the representative mint-derived AgNPs (Figure S4b) show regions of micro-scale agglomerates composed of densely packed, angular subunits, together with a background of smaller scattered entities. To complement the dry-state TEM readout, colloidal properties in aqueous media were evaluated by DLS and electrophoretic mobility ζ-potential (see Figure S5). Mint-derived AgNPs exhibited a smaller average hydrodynamic diameter and lower polydispersity (90 nm; PDI 0.3) than avocado-derived AgNPs (204 nm; PDI 0.5), while avocado-derived AgNPs present a more negative interfacial potential (−30 mV vs. −16 mV).

Taken together, these results indicate that avocado-derived AgNPs behave as the more heterogeneous dispersion by DLS despite their higher-magnitude negative ζ-potential, suggesting that the broader hydrodynamic size distribution is not explained by electrostatics alone and may reflect the contribution of a minor population of larger species and/or a thicker hydrated plant-derived capping layer in dispersion.

These extract-dependent physicochemical fingerprints establish the baseline colloidal state prior to BC loading and are used below to contextualize route-dependent plasmonic organization and antibacterial performance in BC-AgNP membranes.

### 2.3. Synthesis and Characterization of Bacterial Cellulose Membranes

Bacterial cellulose membranes (BC) were synthesized using Komagataeibacter hansenii (ATCC 53582) grown in Hestrin–Schramm (HS) culture medium under static conditions for 7 days. After harvesting and purification, various physicochemical parameters of the membranes were determined. The results obtained are summarized in Table 2.

The physicochemical and mechanical properties summarized in Table 2 are in line with previously reported values for bacterial cellulose membranes produced by *Komagataeibacter hansenii* (ATCC 53582) under static cultivation in Hestrin–Schramm (HS) medium. Parameters such as crystalline index, nanofibril diameter, porosity, and water-holding capacity fall within the typical range described for native BC obtained under comparable conditions. Similarly, the measured thickness and mechanical performance in both dry and hydrated states are consistent with those reported for purified BC membranes. Overall, these findings indicate that the membranes display the expected structural and functional characteristics of BC produced under static HS cultivation [23].

### 2.4. Functionalization of Bacterial Cellulose Membranes with Silver Nanoparticles

Two different strategies were explored to functionalize BC membranes with AgNP. The first strategy consisted of immersing BC membranes in a suspension containing pre-synthesized AgNPs, whereas the second strategy involved the in situ synthesis of AgNPs directly within the bacterial cellulose membrane.

In these assays, AgNP synthesis, either by immersion loading into bacterial cellulose or by in situ formation, was carried out at 23 °C and pH 7, as these conditions yielded higher LSPR peak intensity and narrower bandwidth.

**Figure 1:**
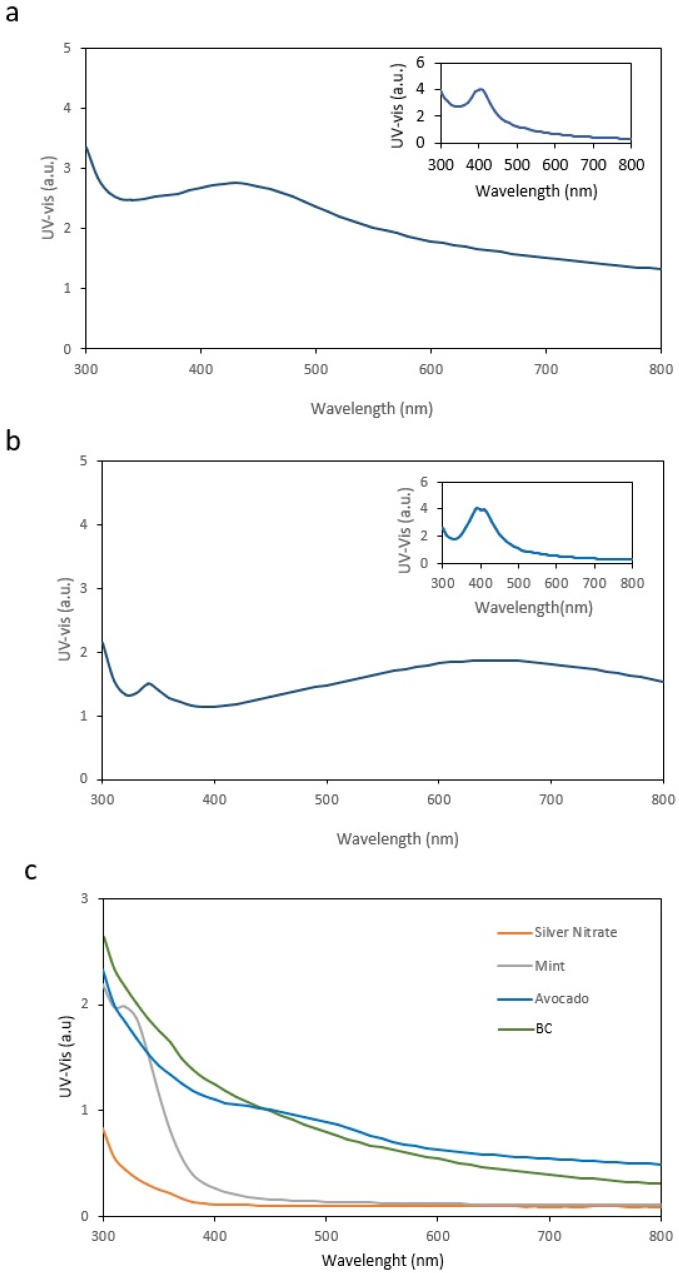
(**a**) UV–Vis spectrum obtained from a BC membrane immersed in an AgNP suspension synthesized by combining 0.01 M AgNO_3_ with avocado extract. The inset shows the corresponding UV–Vis spectrum of the AgNP liquid suspension prepared using the same reagents. (**b**) UV–Vis spectrum obtained from a BC membrane immersed in an AgNP suspension synthesized by combining 0.01 M AgNO_3_ with mint extract. The inset shows the corresponding UV–Vis spectrum of the AgNP liquid suspension prepared using the same reagents. (**c**) UV–Vis spectrum of a pristine BC membrane, showing the absence of an LSPR peak. AgNP synthesis was performed at 23 °C and pH 7.

For functionalization by immersion, BC membranes were submerged in solutions containing AgNPs previously synthesized using either mint or avocado. After immersion, the membranes were removed and each membrane was analyzed by UV–Vis spectroscopy. Figure 1a,b show the UV–Vis spectra of BC membranes loaded with AgNPs derived from avocado and mint extracts, respectively, while the inset panels display the spectra of the corresponding AgNPs in dispersion.

Comparison of the results indicates that, in both cases, there is a clear alteration in the UV–Vis spectral features of the AgNP-loaded BC membranes relative to the corresponding AgNP suspensions. Notably, neither BC membranes, mint extract, avocado extract, nor silver nitrate alone contributed to the UV–Vis peak (Figure 1c). Accordingly, the appearance of a characteristic LSPR band in the spectra of AgNP-loaded BC membranes confirms the presence of AgNPs in the membrane matrix.

The observed changes in the UV–Vis spectra include a decrease in the amplitude of the maximum peak λ_max_ and a noticeable broadening of the peak relative to the liquid suspensions.

**Figure 2:**
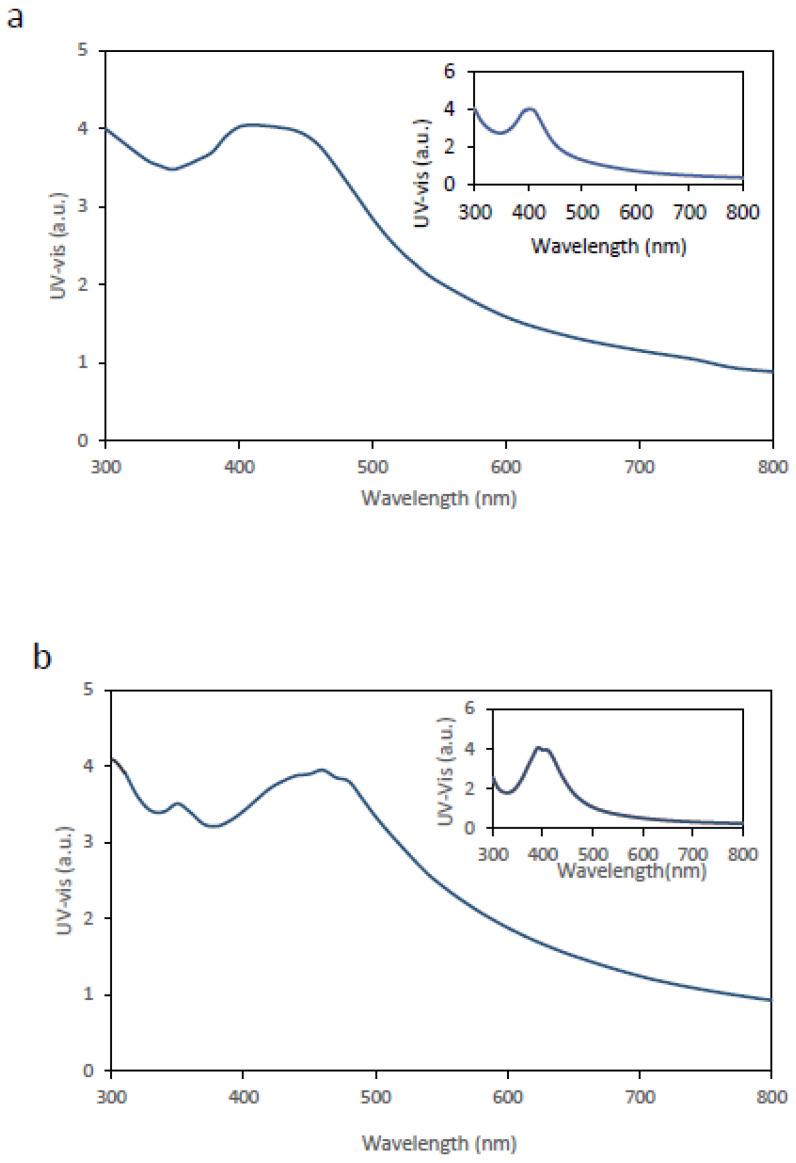
(**a**) UV–Vis spectrum obtained from a BC membrane with AgNP synthesized in situ using 0.01 M AgNO_3_ and avocado extract. (**b**) UV–Vis spectrum obtained from a BC membrane with AgNP synthesized in situ using 0.01 M AgNO_3_ and mint extract. The inset in both panels shows the corresponding UV–Vis spectrum of the AgNP aqueous suspension prepared using the same reagents. AgNP synthesis was performed at 23 °C and pH 7.

The second approach involved the in situ synthesis of AgNPs directly within the BC membrane, and the resulting membranes were characterized by UV–Vis spectroscopy (Figure 2). In both Figure 2a (avocado extract) and Figure 2b (mint extract), a clear improvement in the spectral response is observed, evidenced by increased peak intensity and improved definition of the λ_max_ peak compared with membranes obtained by immersion. The inset panels display the spectra corresponding to dispersed AgNPs.

For both avocado- and mint-mediated syntheses, the UV–Vis spectra obtained from in situ-functionalized BC membranes more closely resemble those of aqueous AgNP suspensions than the spectra obtained from BC membranes functionalized by immersion. Although the membranes exhibit peak intensities broadly comparable to those of AgNPs synthesized in aqueous suspension, a discernible red shift of the λ_max_ peak, together with band broadening, is observed. These spectral features are consistent with the mechanisms discussed above.

Comparison of the UV–Vis spectra of the immersion-loaded membranes (Figure 1a,b) and the in situ-synthesized membranes (Figure 2a,b) indicates that immersion loading yields AgNPs in a more heterogeneous and/or more aggregated state (with a relevant fraction of clusters) within and/or on the BC matrix. This is consistent with plasmonic coupling (red shift), increased damping of the resonance (lower peak intensity), and pronounced band broadening due to polydispersity and microenvironmental heterogeneity. In contrast, in situ synthesis promotes more homogeneous nucleation and improved dispersion/segregation of AgNPs within the fibrillar BC network, resulting in a sharper and more intense LSPR band.

Subsequently, ICP analysis was performed to quantify the silver content in the membranes. The results are summarized in Table 3, where clear differences can be observed depending on the method used for AgNP incorporation into the BC matrix. Specifically, membranes prepared by immersion exhibit a significantly lower silver content than those obtained by in situ synthesis. This behavior can be attributed to the limited ability of pre-synthesized AgNPs to penetrate and diffuse through the dense nanofibrous network of BC. In contrast, during in situ synthesis, the diffusing species are initially AgNO_3_ molecules, followed by the reducing agents present in the plant extracts. These molecular species, being smaller and dissolved in an aqueous medium, can more readily diffuse into the internal structure of the membrane, leading to a more homogeneous distribution and higher overall nanoparticle loading.

Moreover, the in situ formation of AgNPs within the BC network likely promotes stronger interactions between the nanoparticles and the cellulose fibrils, enhancing their retention within the matrix and reducing potential losses during subsequent washing steps. This effect can contribute to the higher silver content observed in membranes synthesized via this method.

It is also noteworthy that, beyond the influence of the incorporation method, a consistently higher nanoparticle content is observed in membranes prepared using avocado seed extract compared to those synthesized with mint extract. This difference is likely related to the distinct chemical composition of the extracts, particularly their content of polyphenols and other reducing and stabilizing agents, which can influence both the reduction efficiency of Ag^+^ ions and the stabilization of the formed nanoparticles.

Finally, the distribution of AgNPs within the different membranes was analyzed by SEM–EDS surface mapping to evaluate the presence and spatial distribution of silver in the BC matrix. The elemental maps (Figure S6) reveal clear differences depending on both the incorporation method and the plant extract used. Membranes loaded by immersion (Figure S6a,b) exhibit a less homogeneous distribution, with localized regions of higher intensity indicative of nanoparticle aggregation and a lower overall signal, suggesting limited incorporation efficiency. In contrast, membranes prepared via in situ synthesis (Figure S6c,d) display a more uniform and continuous distribution, with fewer aggregates and a higher overall intensity, indicating improved dispersion and greater nanoparticle loading within the BC network. Moreover, for both incorporation methods, membranes synthesized using avocado seed extract (Figure S6b,d) show a stronger and more widespread signal than those obtained with mint extract (Figure S6a,c), suggesting a higher nanoparticle content and more effective incorporation.

To further assess nanoparticle penetration, cross-sections of the membranes were analyzed (Figure S7). Again, clear differences are observed depending on the incorporation method. Membranes prepared by immersion (Figure S6a,b) exhibit a lower density and a gradient in nanoparticle distribution across the cross-section, with higher concentrations near the surface that decrease toward the interior. In contrast, membranes obtained by in situ synthesis (Figure S6c,d) show a more uniform distribution throughout the cross-section.

This behavior can be attributed to the same mechanisms previously discussed, i.e., the limited ability of pre-synthesized AgNPs to penetrate the cellulose fiber network, leading to their accumulation near the membrane surface. Conversely, in in situ synthesis, the diffusing species, such as AgNO_3_ and plant-derived reducing agents, are smaller and dissolved in an aqueous medium, which facilitates their transport into the interior of the BC membrane and promotes a more homogeneous nanoparticle distribution.

Overall, these results highlight the critical role of both the synthesis route and the nature of the reducing agent in controlling the distribution and loading of AgNPs within bacterial cellulose membranes. In particular, in situ synthesis within the BC matrix emerges as a more efficient strategy than liquid-phase synthesis followed by nanoparticle adsorption or uptake.

### 2.5. AgNP Release from BC Membranes

To further verify the presence of a nanoparticulate fraction associated with the membrane, BC-AgNP samples were incubated in Milli-Q water under agitation at room temperature for 24 h and the resulting suspensions were analyzed by DLS and ζ-potential. DLS detected nanoparticle-sized populations in all release suspensions (mean hydrodynamic diameters in the 85–283 nm range), confirming that a nanoparticulate fraction was recovered from the membranes under these conditions (see Figures S8 and S9).

Released NPs from in situ-functionalized membranes showed smaller hydrodynamic diameters than those released from immersion-loaded membranes for both extracts. Specifically, mint-derived AgNP-BC (in situ) released NPs with an average hydrodynamic diameter of 85 nm (PDI 0.5; ζ = −15 mV), whereas avocado-derived AgNP-BC (in situ) released NPs with an average hydrodynamic diameter of 127 nm (PDI 0.3; ζ = −12 mV). In contrast, NPs released from immersion-loaded membranes were larger by DLS (Mint@BC immersed: 159 nm, PDI 0.3, ζ = −17 mV; Avo@BC immersed: 283 nm, PDI 0.4, ζ = −29 mV). Overall, these measurements confirm that the hydrodynamic characteristics of the released nanoparticulate fraction depend on the membrane functionalization route and on the plant extract used for synthesis.

Notably, the released fraction from Mint@BC (in situ) displayed a mean hydrodynamic diameter close to that of the corresponding as-synthesized colloid (90 nm), whereas immersion-derived released fractions were shifted to larger hydrodynamic sizes.

### 2.6. Analysis of the Antibacterial Activity of BC Membranes Functionalized with AgNPs

Next, the antibacterial efficacy of bacterial cellulose membranes functionalized with silver nanoparticles (BC-AgNPs) was investigated. *Escherichia coli* TOP10 constitutively expressing green fluorescent protein (GFP) (*E. coli*:GFP) was used as the model organism.

A series of assays were conducted to evaluate antimicrobial performance. In the first assay, the diameter of the bacterial growth inhibition zone was measured on Petri dishes containing a uniformly distributed *E. coli*:GFP lawn, onto which circular BC-AgNP membranes (4 mm in diameter) were placed.

**Figure 3:**
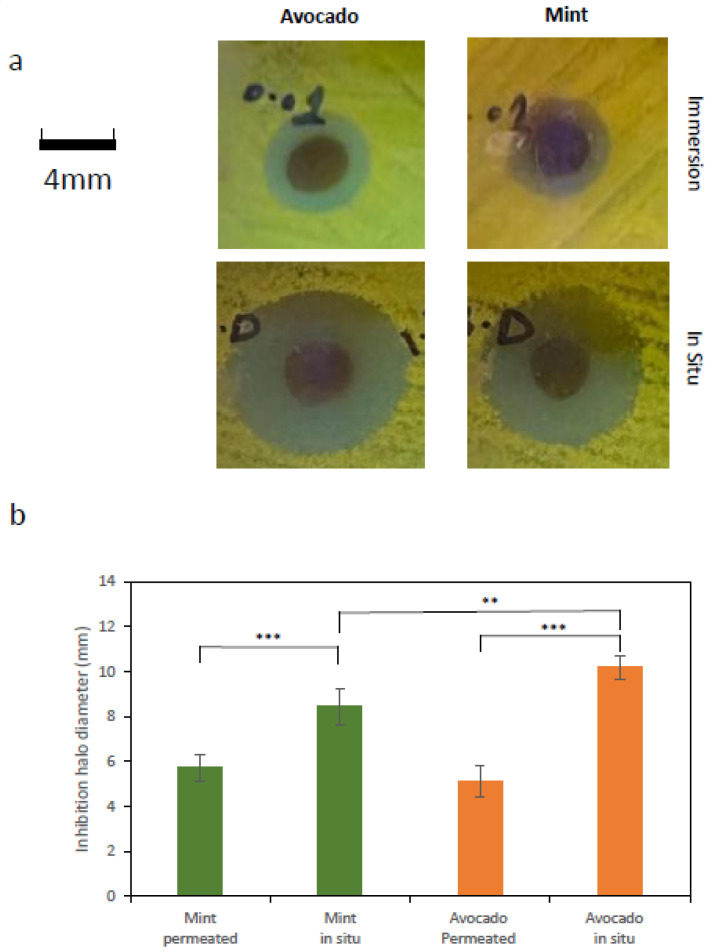
(**a**) Representative images of characteristic growth inhibition halos generated by BC–AgNP membranes prepared either by immersion or by in situ synthesis using avocado or mint extracts. The membranes were placed on Petri dishes containing LB agar uniformly inoculated with an *E. coli*–GFP liquid culture, with five BC-AgNP membranes positioned on each plate. A statistically significant difference was observed in the size of the inhibition halos produced by BC–AgNP synthesized in situ using avocado extract compared with those synthesized using mint extract, with the avocado extract-derived BC-AgNPs exhibiting higher antibacterial efficiency. Statistically significant differences are indicated as ** *p* < 0.01, *** *p* < 0.001. (**b**) Average diameter of the bacterial growth-inhibition halos measured on the Petri dishes. Values represent the mean of five independent experiments, and error bars correspond to the standard deviation.

The bacterial growth-inhibition capacity of membrane pieces functionalized either by immersion or by in situ AgNP synthesis was assessed. Inhibitory efficiency was quantified by measuring the diameter of the inhibition zones observed on the agar plates. For illustrative purposes, Figure 3a shows representative images of characteristic inhibition zones generated by BC-AgNP membranes prepared using avocado and mint extracts for nanoparticle synthesis. The image shows a clear difference in the size of the inhibition zones generated by membranes functionalized by immersion, which are markedly smaller than those corresponding to membranes functionalized via in situ AgNP synthesis. This comparison clearly demonstrates that the latter approach produces membranes that are substantially more effective in terms of antibacterial activity.

Figure 3b reports the inhibition zone diameters under the different tested conditions. As shown, antibacterial efficiency was consistently higher for membranes functionalized via in situ synthesis, regardless of whether mint or avocado extract was used.

Notably, a statistically significant difference was also observed in the size of the inhibition areas produced by BC-AgNPs synthesized in situ using avocado extract compared with those synthesized using mint extract, with avocado extract-derived BC-AgNPs exhibiting higher antibacterial efficiency (Welch’s *t*-test, *p* < 0.001).

Subsequently, the bactericidal activity of the nanoparticles was evaluated. To this end, two different assays were performed using BC-AgNP membranes functionalized via in situ synthesis with either avocado or mint extracts, as these formulations showed the highest performance in the previous assays.

**Figure 4:**
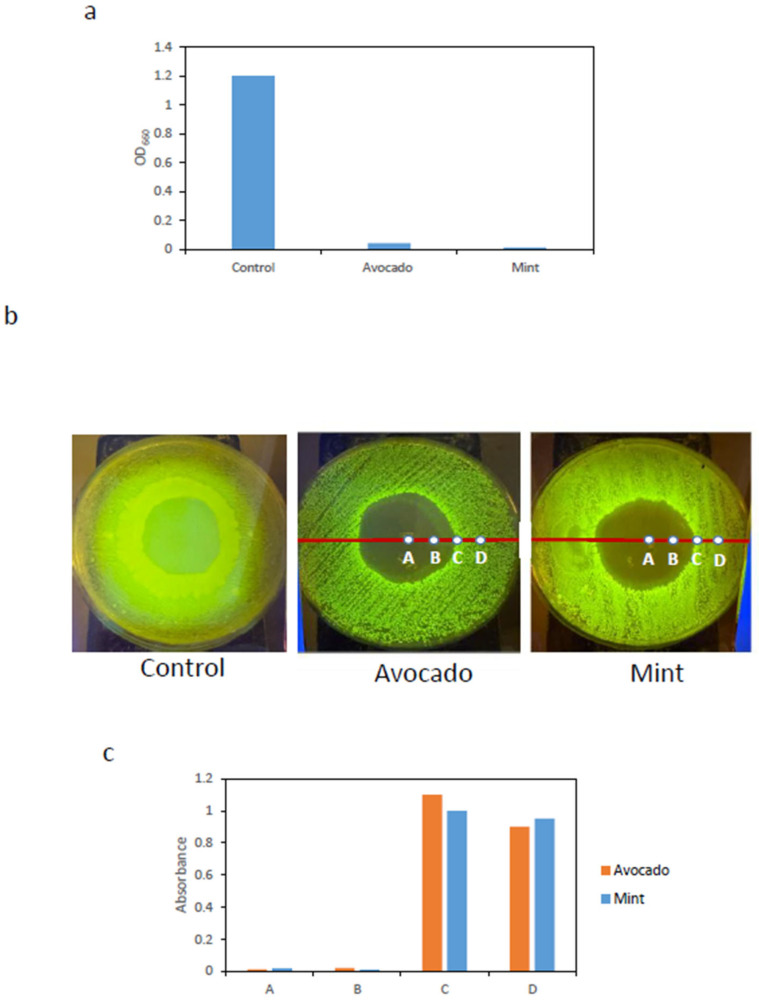
(**a**) Absorbance measurements of liquid cultures of *E. coli*–GFP in the presence of BC- AgNP membranes. A pristine BC membrane without AgNP was used as a control. Only the control exhibited significant bacterial growth, whereas no growth was detected in the presence of BC-AgNP membranes synthesized using either avocado or mint extracts. (**b**) Representative images of areas lacking observable bacterial growth, visualized under a transilluminator, generated by the presence of BC-AgNP membranes. In the control condition, where a BC membrane without AgNPs was used, no bacteria-free area was observed. Letters A, B, C, and D indicate the positions from which surface samples were collected for subsequent inoculation of liquid cultures. (**c**) Absorbance measurements of liquid cultures inoculated with samples collected from positions A, B, C, and D. No bacterial growth was observed in cultures inoculated with samples from points A and B, indicating that these samples did not contain viable bacteria, whereas efficient bacterial growth was observed for samples from points C and D.

The first assay was carried out in liquid culture, in which circular BC-AgNP samples (4 mm in diameter) were introduced into *E. coli*:GFP cultures. After incubation, the absorbance of the resulting cultures was measured at 660 nm. Figure 4a shows that significant bacterial growth was observed only in the control culture, whereas no growth was detected in cultures exposed to BC-AgNP membranes, indicating that these membranes exhibit strong bactericidal activity.

The second assay was performed on solid surfaces. Circular BC-AgNP membranes (30 mm in diameter), synthesized in situ using either avocado or mint extracts, were placed onto LB agar plates that had been previously inoculated with a uniformly spread *E.coli*:GFP liquid culture.

After incubation, the membranes were removed, and clear zones with no observable bacterial growth were evident upon transillumination of the plates. In contrast, the control condition, consisting of a BC membrane immersed in AgNO_3_ and subsequently washed, but not exposed to plant extracts, exhibited GFP fluorescence across the entire plate surface. Figure 4b shows representative images highlighting areas lacking detectable bacterial growth. Starting from the center of the growth inhibition zone, surface samples were collected at four positions (points A, B, C, and D in Figure 4b). Each sample was transferred to LB medium and incubated. Subsequently, absorbance was measured at 660 nm.

As shown in Figure 4c, samples collected from points A and B, located within the zone where no bacterial growth was observed, exhibited very low absorbance values, indicating the absence of bacterial growth in liquid culture. In contrast, samples from points C and D showed high absorbance values, indicating clear bacterial growth. These results, consistent with the previous assay, further confirm the strong bactericidal activity of the BC-AgNP membranes.

## 3. Discussion

The results presented in this work highlight the strong interdependence between synthesis conditions, nanoparticle incorporation strategy, and the antibacterial performance of BC membranes functionalized with green-synthesized AgNPs. In aqueous-phase synthesis, both avocado seed and mint extracts enabled AgNP formation; however, their properties were strongly influenced by extract composition. Compositional analyses showed a lower flavonoid content and a higher carbohydrate content in the avocado extract compared with the mint extract (Table 1). Consistent with this compositional difference, aqueous AgNPs showed extract-dependent hydrodynamic signatures, with higher polydispersity for avocado-derived AgNPs than for mint-derived AgNPs.

Beyond extract composition, pH and temperature critically affect formation kinetics and the physicochemical properties of AgNPs. Increasing temperature accelerates nanoparticle formation by enhancing reaction kinetics, whereas extreme pH values can alter reduction pathways, modulate nucleation and growth processes, and compromise colloidal stability, ultimately impacting particle size, morphology, and dispersion [24]. Under our conditions, neutral pH (pH 7) and moderate temperature (23 °C) were optimal, yielding well-defined and relatively narrow LSPR bands in UV-Vis spectra, indicative of efficient nanoparticle formation with a comparatively narrow size distribution. In contrast, acidic conditions markedly hindered synthesis, likely due to protonation of functional groups (phenolics, carboxylates), which reduces their reducing and capping capability, in agreement with previous reports [25]. At elevated temperatures, although reaction kinetics are accelerated, broader LSPR peaks suggest less controlled growth, increased polydispersity and/or aggregation, consistent with thermal destabilization of capping interactions.

The formation of AgNPs using mint and avocado seed extracts at pH 7 and 23 °C was confirmed by TEM. Avocado-derived AgNPs exhibit a heterogeneous population, where individual nanoparticles coexist with small clusters and occasional larger aggregates, with predominantly rounded to slightly faceted morphologies. In contrast, mint-derived AgNPs show regions of micro-scale agglomerates composed of densely packed angular subunits, together with smaller dispersed particles.

These TEM observations were complemented by DLS analysis, which provides information on the hydrodynamic diameter of nanoparticles in suspension, including the contribution of solvation layers, capping agents, and dynamic aggregates. In agreement with TEM, avocado-derived AgNPs display larger hydrodynamic sizes and higher polydispersity than mint-derived systems, reflecting the presence of aggregates. However, the magnitude of this increase also suggests additional contributions from a thicker organic corona or transient interparticle associations in solution.

Despite exhibiting a more negative ζ-potential, typically associated with higher colloidal stability, avocado-derived AgNPs show greater heterogeneity in dispersion. This indicates that electrostatic stabilization alone does not fully govern their behavior, and that steric effects from plant-derived capping agents, as well as the presence of larger aggregates, play an important role. Overall, the broader hydrodynamic size distribution of avocado-derived AgNPs arises from the combined influence of structural heterogeneity and solution-phase effects.

A central aspect of this study is the comparison of two membrane functionalization strategies: (i) immersion of BC membranes in suspensions of pre-formed AgNPs and (ii) in situ synthesis of AgNPs within the BC matrix. In both approaches, nanoparticle presence and organization were assessed via the LSPR band in UV–Vis spectra, which provides insight into particle size, morphology, and aggregation state. Immersion-loaded membranes displayed reduced peak intensity and pronounced band broadening relative to aqueous suspensions, consistent with limited nanoparticle penetration, heterogeneous local environments, and enhanced NP–NP interactions within the BC network. In contrast, in situ synthesis produced more intense and better-defined LSPR features, indicating more efficient nanoparticle incorporation and a more homogeneous spatial distribution.

These trends are consistent with ICP results (Table 3). Avocado-derived systems exhibited higher silver content than mint-derived ones. SEM–EDS mapping further supports these findings, revealing a more uniform and intense Ag signal for in situ membranes and a higher nanoparticle content in avocado-based systems. Together, these results demonstrate that in situ synthesis and avocado extract promote enhanced nanoparticle loading within the BC matrix.

Antibacterial assays functionally validate these differences: BC–AgNP membranes prepared via in situ synthesis showed higher efficacy against *E. coli* than immersion-functionalized membranes, as evidenced by larger inhibition halos on solid media. Additional experiments confirmed a bactericidal, rather than merely bacteriostatic, effect: no regrowth was observed after membrane removal and transfer to fresh medium, and sampling from within inhibition zones yielded no detectable growth upon re-culturing, whereas samples collected outside inhibition zones showed robust growth. This behavior is consistent with well-established antibacterial mechanisms of AgNPs, including Ag^+^ release, membrane damage, protein/enzyme denaturation, ROS generation, and interference with DNA replication and protein synthesis [26,27]. In our system, AgNPs embedded in BC likely act as a local Ag^+^ source and facilitate direct nanoparticle–bacteria contact, accounting for the observed inhibition.

Although ICP analysis revealed differences in silver content among the membranes, antibacterial activity cannot be directly correlated with the total amount of silver alone. The antimicrobial performance of AgNP-loaded BC membranes is influenced by several factors, including nanoparticle size, distribution within the matrix, and the release of bioactive Ag^+^ ions. In this context, SEM–EDS analysis suggests that membranes prepared via in situ synthesis exhibit a more homogeneous nanoparticle distribution, whereas membranes prepared by immersion tend to show a more heterogeneous distribution, with evidence of nanoparticle aggregation, which could limit silver ion availability.

Accordingly, the enhanced antibacterial performance observed for in situ-functionalized membranes may be related to their higher nanoparticle loading and more uniform spatial distribution within the BC matrix. As indicated by ICP and SEM–EDS analyses, in situ synthesis appears to promote more efficient incorporation of AgNPs and a more homogeneous dispersion, with fewer localized aggregates compared to immersion-loaded membranes. This improved distribution could increase the effective surface area of accessible nanoparticles and facilitate a more consistent release of Ag^+^ ions, which are known to contribute to antibacterial activity. In contrast, immersion-loaded membranes, characterized by lower nanoparticle content and a more heterogeneous distribution, may present reduced availability of active sites. Overall, these structural differences likely contribute to the improved antibacterial behavior observed for in situ systems, although additional factors may also play a role.

To further substantiate the presence of a nanoparticulate fraction associated with the membranes and to compare route-dependent release behavior, a fraction was recovered by agitation in water and analyzed by DLS and ζ-potential measurements. DLS confirmed the presence of nanoparticle-sized populations in all release suspensions, with mean hydrodynamic diameters in the 85–283 nm range. Notably, particles released from in situ-functionalized membranes exhibited smaller hydrodynamic diameters than those from immersion-loaded membranes for both extracts (mint in situ: 85 nm, PDI 0.5, ζ = −15 mV; avocado in situ: 127 nm, PDI 0.3, ζ = −12 mV; mint immersed: 159 nm, PDI 0.3, ζ = −17 mV; avocado immersed: 283 nm, PDI 0.4, ζ = −29 mV).

Importantly, within the in situ group, where avocado-derived membranes produce larger inhibition halos than mint-derived membranes, the ζ-potential of the released fraction is not more negative for avocado than for mint. Therefore, colloidal stability inferred from ζ-potential alone does not explain the observed differences in antibacterial activity. A mechanistic interpretation would require quantitative release metrics, such as total silver release and/or Ag^+^ flux, which were not determined in this study. Consequently, while higher silver loading and improved nanoparticle distribution likely contribute to the observed antibacterial performance, understanding the underlying mechanisms would require quantitative release metrics (e.g., total Ag and/or Ag^+^ flux), which were not measured here.

Finally, while both functionalization strategies yield distinct plasmonic profiles, their suitability may depend on the intended application. Red/NIR-shifted features associated with plasmonic coupling may be advantageous for photothermal or sensing applications [28,29,30,31,32], whereas in situ synthesis provides superior antibacterial performance under the conditions tested. These findings are consistent with previous reports on BC-based nanocomposites, where nanoparticle incorporation enhances antimicrobial activity through combined contact and release mechanisms [33,34].

## 4. Materials and Methods Reagents Used

The plant materials, avocado seeds and mint leaves, were sourced locally from Barcelona, Spain. Silver nitrate (≥99% purity) was procured from Thermo Fisher Scientific, Waltham, MA, USA and served as the silver-ion precursor. To adjust the pH during nanoparticle synthesis, 0.1 M NaOH and 0.1 M HCl solutions were prepared, enabling control over the reaction environment. A 0.1% (*w*/*v*) sodium citrate solution (Sigma-Aldrich, St. Louis, MO, USA) was added to the reaction mixture as a stabilizer to prevent nanoparticle agglomeration and promote colloidal stability.

### 4.1. Preparation of Bacterial Cellulose Membranes

Bacterial cellulose (BC) membranes were prepared using the acetic-acid bacterium *Komagataeibacter hansenii* (ATCC 53582), a well-established model organism for BC biosynthesis [35]. The process began by preparing a liquid-phase inoculum: the strain was cultured in Hestrin–Schramm (HS) medium (glucose 20 g L^−1^, yeast extract 5 g L^−1^, peptone 5 g L^−1^, sodium phosphate dibasic 2.7 g L^−1^, citric acid 1.1 g L^−1^) at an initial pH of ~5.5. The preculture was incubated at 30 °C under orbital agitation at 200 rpm for 3 days to ensure high cell density.

Following preculture, sterile trays containing 250 mL of fresh HS medium were inoculated with 20% (*v*/*v*) of the liquid culture. The trays were then incubated under static conditions at room temperature, allowing BC pellicles to progressively form at the air–liquid interface for 7 days. Upon completion of the cultivation period, BC membranes were harvested and purified by immersion in a 0.1 M NaOH solution at 80 °C for 30 min to remove bacterial cells and associated impurities, e.g., proteins and DNA, in accordance with standard procedures. Following the alkaline treatment, membranes were thoroughly rinsed multiple times with distilled water until pH ≈ 7 was achieved.

### 4.2. Avocado Seed Extract

Avocado seeds were collected, finely chopped, and transferred into a beaker containing distilled water. The mixture was heated at 50 °C for 40 min to facilitate the extraction of reducing phytochemicals (e.g., phenolics, flavonoids, and polysaccharides), in accordance with previously published protocols [36]. After cooling to room temperature, the mixture was vacuum-filtered using an MF-Millipore^TM^ filter (Merck Millipore, Darmstadt, Germany) with a 5 µm pore size to remove solid debris. The resulting filtrate constituted the avocado seed extract, which was stored in airtight containers at −20 °C until use.

### 4.3. Mint Leaf Extract

Fresh mint leaves (0.5 g) were thoroughly washed with distilled water to remove surface impurities. The cleaned leaves were incubated in a known volume of distilled water and heated, for instance, boiled until 70 mL remained from an initial 100 mL, in accordance with common protocols [37]. After heating, the extract was cooled to room temperature and vacuum-filtered using an MF-Millipore™ filter with a 5 µm pore size. The clear filtrate was aliquoted and stored at −20 °C for subsequent nanoparticle synthesis.

### 4.4. Determination of Extract Composition

#### 4.4.1. Determination of Total Phenolic Content (TPC)

Total phenolic content (TPC) was determined using the Folin–Ciocalteu colorimetric method, following previously published protocols [38]. An aliquot of 0.5 mL of the extract was mixed with 2.5 mL of 0.2 M Folin–Ciocalteu reagent solution (Sigma-Aldrich, USA). The mixture was allowed to stand for 5 min, after which 2 mL of 7.5% (*w*/*v*) sodium carbonate (Sigma-Aldrich, USA) was added. The reaction mixture was incubated for 15 min at 40 °C, and absorbance was measured at 760 nm using a UV–Vis spectrophotometer.

A calibration curve was constructed using gallic acid (Sigma-Aldrich, USA) standard solutions (10–200 mg/L). Total phenolic content was expressed as mg gallic acid equivalents per gram of dry extract (mg GAE/g dry extract).

#### 4.4.2. Determination of Total Flavonoid Content (TFC)

Total flavonoid content (TFC) was determined using the aluminum chloride (AlCl_3_) colorimetric assay, following previously described methods [39]. The dry extract was dissolved in methanol to a concentration of 1 mg/mL. An aliquot of 0.5 mL of the extract solution was mixed with 2.5 mL of distilled water, followed by the addition of 150 µL of 5% (*w*/*v*) sodium nitrite (Sigma-Aldrich, USA). After 5 min of incubation at room temperature, 150 µL of 10% (*w*/*v*) aluminum chloride (Sigma-Aldrich, USA) solution was added. After an additional 6 min, 1 mL of 1 M sodium hydroxide (Sigma-Aldrich, USA) was added, and the reaction mixture was thoroughly mixed. Absorbance was measured at 510 nm using a UV–Vis spectrophotometer.

Quercetin (Sigma-Aldrich, USA) standard solutions (0–100 mg/L) were used to construct the calibration curve, and results were expressed as mg quercetin equivalents per g of dry extract (mg QE/g dry extract).

#### 4.4.3. Determination of Total Carbohydrates

Total carbohydrate content was determined using the phenol–sulfuric acid method, following previously described methods [40]. The dry extract was dissolved in distilled water, and aliquots were prepared in test tubes to a final volume of 2.0 mL with distilled water. Glucose (Sigma-Aldrich, USA) was used as the reference compound for calibration.

To each tube, 50 µL of phenol solution in water (80% *w*/*v*) (Sigma-Aldrich, USA) was added, followed by the rapid addition of 5.0 mL of concentrated sulfuric acid (95–98%) (Sigma-Aldrich, USA). The sulfuric acid was added directly to the liquid surface to ensure proper mixing and heat generation. The reaction mixtures were vortexed and allowed to stand for 10 min, after which the tubes were placed in a 25 °C water bath for 10 min to allow color development and stabilization.

Absorbance was measured at 490 nm using a UV–Vis spectrophotometer. A calibration curve was constructed using glucose standard solutions, and total carbohydrate content was expressed as mg glucose equivalents per g of dry extract (mg GE/g dry extract).

### 4.5. Characterization of Bacterial Cellulose Membranes

In order to characterize the main properties of the BC membranes, different techniques were employed.

To determine the crystallinity index, X-ray diffraction (XRD) analysis was performed using a Bruker D8 Advance diffractometer (Bruker Corporation, Billerica, MA, USA) equipped with Cu Kα radiation (λ = 1.5406 Å), operating at 40 kV and 40 mA. Diffraction patterns were recorded over a 2θ range of 10–40° using an appropriate step size and scanning speed. The crystallinity index (CrI) was calculated according to the Segal method [41], based on the intensity of the main crystalline peak and the minimum intensity corresponding to the amorphous region.

The nanofibril diameter was analyzed by scanning electron microscopy (SEM) using a JEOL JSM-IT500 (JEOL Ltd., Tokyo, Japan). Fibril diameters were measured from representative micrographs using image analysis software (ImageJ, version 1.54s NIH, Bethesda, MD, USA).

Porosity in the hydrated state and water-holding capacity (WHC) were determined gravimetrically. For porosity estimation, membranes were weighed in the fully hydrated state and after drying at room temperature to a constant weight. WHC was calculated as the percentage of retained water relative to the total hydrated mass.

Membrane thickness was measured in the hydrated state using a digital micrometer at multiple positions, and the average value was calculated.

Mechanical properties, including tensile strength (under dry and wet conditions) and Young’s modulus (dry state), were evaluated using a universal testing machine (UTM). The samples were cut into standardized strips and tested at a constant strain rate. For wet measurements, the membranes were equilibrated in distilled water prior to testing.

### 4.6. Green Synthesis of Silver Nanoparticles Using Mint and Avocado Extracts in Aqueous Media

Silver nitrate (AgNO_3_) was dissolved in Milli-Q water to prepare precursor solutions at varying concentrations (0.01, 0.0075, 0.005, 0.002 and 0.001 M). These concentrations allowed control over nanoparticle nucleation density and size, consistent with literature indicating that higher AgNO_3_ concentrations often increase nucleation rates and can shift the LSPR peak position depending on growth and aggregation behavior [42].

To explore the influence of the reaction environment on nanoparticle formation, syntheses were conducted at three temperatures (4 °C, 23 °C and 70 °C) and under three pH conditions (acidic pH = 5, neutral pH = 7 and basic pH = 11), adjusted using 0.1 M HCl or NaOH.

For low-temperature synthesis at 4 °C, reactions were carried out in an ice bath. Plant extracts were added dropwise to the AgNO_3_ solution at a 1:1 volume ratio to promote controlled nucleation. After mixing, the reaction mixture was incubated at 4 °C with gentle shaking at 120 rpm for 24 h.

For room and elevated temperatures (23 °C and 70 °C), the same 1:1 extract-to-AgNO_3_ ratio was used, and reaction mixtures were incubated at the respective temperatures with shaking at 120 rpm for 24 h.

In all syntheses, the concentration of the plant extract was kept constant while the AgNO_3_ concentration was varied. Additionally, 0.1% (*w*/*v*) sodium citrate solution was included as a stabilizing agent to prevent nanoparticle agglomeration. Citrate is a well-defined capping agent that adsorbs strongly to the nanoparticle surface, providing consistent electrostatic stabilization and limiting uncontrolled growth or aggregation. Its use complements the variable stabilizing effects of extract components, helping to achieve more controlled nanoparticle formation across different synthesis conditions [43,44].

Verification of AgNP formation relied initially on the characteristic color change of the reaction mixture, which is a qualitative indicator of nanoparticle formation associated with localized surface plasmon resonance (LSPR). UV–Vis spectroscopy was subsequently employed to monitor LSPR features and shifts, which can reflect changes in nanoparticle size distribution and aggregation state [45,46].

### 4.7. Functionalization of Bacterial Cellulose Membranes by Immersion in Aqueous Silver Nanoparticle Suspensions

For the preparation of membranes functionalized with silver nanoparticles, BC membranes with standardized dimensions were immersed in aqueous suspensions containing AgNPs, which were previously synthesized using mint and avocado seed extracts, respectively. The membranes were maintained in the AgNP suspensions under agitation at 200 rpm for 24 h at room temperature. Subsequently, the membranes were retrieved and washed with Milli-Q water to remove any residual nanoparticles not incorporated into the BC matrix.

### 4.8. In Situ Synthesis of Silver Nanoparticles Within Bacterial Cellulose Membranes Using Plant Extracts (Mint and Avocado)

BC membranes with standardized dimensions were sectioned and immersed in 10 mL of AgNO_3_ solutions at different concentrations (0.01, 0.0075, 0.005, 0.002, and 0.001 M) for 24 h to facilitate the diffusion of Ag^+^ ions into the porous BC network and promote homogeneous precursor distribution.

After loading, the membranes were collected; excess AgNO_3_ was removed by pressing the membranes between two sheets of paper, and they were then immersed in 100 mL of either mint or avocado extract and incubated for an additional 24 h. This step enabled biomolecules present in the extracts, e.g., phenolics and flavonoids, to reduce Ag^+^ ions within the BC matrix. A visible color change of the membranes, attributable to LSPR, served as an immediate indicator of AgNP formation.

Upon completion of the synthesis, the membranes were thoroughly rinsed three times with abundant Milli-Q water to remove residual reagents and unbound silver ions, ensuring clean nanocomposite surfaces for subsequent characterization and applications.

### 4.9. Characterization of AgNPs

#### 4.9.1. Characterization of AgNPs by UV–Visible Spectroscopy

The synthesized silver nanoparticles were characterized by UV–Visible (UV–Vis) absorption spectroscopy. Spectral data were collected in the 300–800 nm range using a multimode microplate reader (BioTek Synergy HTX, Winooski, VT, USA), which employs a monochromator-based absorbance system capable of wavelength scanning in 1 nm increments.

#### 4.9.2. Characterization of AgNPs by Transmission Electron Microscopy (TEM)

Transmission electron microscopy (TEM) analysis was carried out with a JEOL JEM 1010 microscope at 80 kV. Samples were dispersed in ethanol, applied to copper grids (Formvar/carbon-supported copper grids, 200 mesh (Ted Pella Inc., Redding, CA, USA), and dried overnight at room temperature.

#### 4.9.3. Characterization of AgNPs by Dynamic Light Scattering (DLS)

Hydrodynamic diameter and zeta potential were measured using a Zetasizer Nano ZS (Malvern Instruments, Worcestershire, UK). The samples were diluted in water. Disposable cuvettes (Sigma-Aldrich, Darmstadt, Germany) were used for size measurements, and folded capillary cells (Malvern Instruments, UK) for zeta potential. The refractive index was 0.2, and the absorption was 3.32. Each sample was measured three times with 10 runs per measurement.

### 4.10. Characterization of BC Membranes Loaded with AgNPs

#### 4.10.1. Inductively Coupled Plasma (ICP) Analysis of BC-AgNP Membranes

To quantify the silver retained in the BC membranes, the samples were digested with a mixture of HCl and HNO_3_ in a microwave oven, following the procedure described by Ashoka et al. [47]. Silver was then analyzed in the resulting solutions by ICP using Jobin Yvon 70 Plus instrument (Horiba Jobin Yvon, Longjumeau, France).

#### 4.10.2. SEM–EDS Characterization of BC Membranes Loaded with AgNPs

Scanning electron microscopy coupled with energy-dispersive X-ray spectroscopy (SEM–EDS) was employed to analyze the morphology and elemental composition of the membranes. The samples were examined using a field-emission scanning electron microscope (FE-SEM, FEI Quanta 250. FEI Company, Hillsboro, OR, USA) equipped with an EDS detector (Oxford Instruments, Abingdon, UK). Prior to analysis, the membranes were sputter-coated with a thin layer of gold. SEM images were acquired at an accelerating voltage of 10–15 kV. Elemental mapping was performed to evaluate the spatial distribution of AgNP within the BC matrix.

### 4.11. Strains Used for Antibacterial Activity Assays

To evaluate the antibacterial activity of BC membranes functionalized with silver nanoparticles (AgNPs), *Escherichia coli* TOP10 (Invitrogen, Carlsbad, CA, USA) was used as the model organism. To facilitate visualization of bacterial colonies, a recombinant strain constitutively expressing green fluorescent protein (GFP) under the control of the *Ptet* promoter [48] was generated.

The genetic constructs were assembled using the BioBrick assembly method (Ginkgo Bioworks, Boston, MA, USA) [49]. The constructs were cloned into the pSB1AK3 backbone (a high-copy plasmid conferring ampicillin resistance). All transformations were performed using chemically competent cells, and the integrity of the genetic constructs was confirmed by Sanger sequencing.

Bacterial cultures were initiated from single colonies obtained from streaked glycerol stocks and inoculated into fresh Lysogeny Broth (LB) supplemented with ampicillin (35 µg mL^−1^; Sigma-Aldrich, St. Louis, MO, USA). Cultures were incubated at 37 °C for 24 h. For long-term storage, bacterial strains were preserved at −80 °C in LB supplemented with 20% (*v*/*v*) glycerol.

### 4.12. Antibacterial Activity Assays of BC Membranes Functionalized with AgNPs

A series of assays were conducted to evaluate the antibacterial activity of BC-AgNP membranes under both solid-surface and liquid conditions. The first assay involved measuring the diameter of the bacterial growth inhibition zone on Petri dishes containing Lysogeny Broth (LB) solidified with 1% agar (LB agar) supplemented with ampicillin. For these assays, overnight *E.coli*:GFP cultures were prepared by inoculating single colonies obtained from streaked glycerol stocks into 5 mL of LB medium. Cultures were incubated at 37 °C with orbital shaking at 200 rpm. Subsequently, 100 µL of culture was uniformly spread over the surface of an LB agar plate. Circular pieces of BC-AgNP membranes were placed at various positions on the plate surface, and plates were incubated at 37 °C for 24 h.

The second assay was carried out in liquid culture. Circular BC-AgNP samples (4 mm in diameter) were introduced into 5 mL of LB medium, which was subsequently inoculated with *E. coli*:GFP from a single colony obtained from streaked glycerol stocks. A BC membrane without nanoparticles was used as a control. Cultures were incubated at 37 °C with orbital shaking at 200 rpm for 24 h. After incubation, BC membranes were removed from the cultures. Subsequently, 1 µL from each culture was transferred into 5 mL of fresh LB medium and incubated again at 37 °C for 24 h with orbital shaking at 200 rpm. The absorbance of the resulting cultures was measured at 660 nm.

## 5. Conclusions

This study demonstrates that synthesis conditions, incorporation strategy, and extract composition critically determine the physicochemical properties and functional performance of BC-AgNP membranes. Among the evaluated approaches, in situ synthesis consistently enabled higher nanoparticle loading, a more homogeneous spatial distribution, and improved antibacterial activity compared to immersion-based loading. Additionally, the use of avocado seed extract led to higher nanoparticle incorporation than mint extract, highlighting the importance of extract composition and reducing-agent chemistry in green synthesis routes.

The combined analysis by UV–Vis spectroscopy, DLS, ICP, SEM–EDS, and antibacterial assays establishes a clear structure–function relationship in which enhanced nanoparticle incorporation and dispersion are associated with improved bactericidal performance. In particular, the more uniform distribution and higher availability of AgNPs in in situ-functionalized membranes likely promote more effective contact- and release-mediated antibacterial mechanisms. Although parameters such as nanoparticle release kinetics and Ag^+^ flux were not quantitatively addressed in this study, the observed trends strongly suggest that these factors contribute to the superior performance of in situ systems.

Furthermore, this work highlights the relevance of green synthesis conditions not only in terms of sustainability but also as a key factor in controlling nanoparticle characteristics and their integration into biopolymer matrices. The ability to tailor nanoparticle formation and distribution through mild conditions (neutral pH and moderate temperature) and natural extracts provides a versatile platform for designing functional materials with tunable properties.

Overall, the results reinforce the potential of bacterial cellulose as a robust and adaptable scaffold for the development of antimicrobial nanocomposites. The combination of BC with in situ green synthesis of AgNPs represents an effective strategy to achieve high nanoparticle loading, controlled dispersion, and strong antibacterial activity. These findings provide valuable insights for the rational design and optimization of next-generation BC-based materials, with potential applications in biomedical devices, wound dressings, and antimicrobial coatings.

## Figures and Tables

**Table 1:** Total phenolic content (TPC), total flavonoid content (TFC), and total carbohydrates in mint leaf and avocado seed extracts.

	Mint Leaf Extract	Avocado Seed Extract	
Component	Content	Content	Method
Total phenolic content (TPC)	93 ± 5 mg GAE/g dry extract	98 ± 6 mg GAE/g dry extract	Folin–Ciocalteu
Total flavonoid content (TFC)	25 ± 4 mg QE/g dry extract	9 ± 4 mg QE/g dry extract	Colorimetric
Total carbohydrates	210 ± 60 mg GE/g	380 ± 84 mg GE/g	Phenol–sulfuric

**Table 2:** Bacterial cellulose parameters.

Property	Value
Crystallinity index (XRD)	72%
Nanofibril diameter	40–90 nm
Porosity	96%
Water-holding capacity (WHC)	98% (*w*/*w*)
Membrane thickness	2.1 mm (wet) 0.2 mm (dry)
Tensile strength (dry)	137 MPa
Young’s modulus (dry)	6 GPa
Tensile strength (wet)	5 MPa

**Table 3:** Silver content.

Incorporation Method	Extract	μg Ag/g BC
Immersion	Mint	17
	Avocado seed	22
In situ	Mint	57
	Avocado seed	68

## Data Availability

The original contributions presented in this study are included in the article/Supplementary Materials. Further inquiries can be directed to the corresponding author.

## Supplementary Materials

The following supporting information can be downloaded at https://www.mdpi.com/article/10.3390/ijms27093943/s1.
