# Supplementary material for: Bacterial Cellulose Membranes Functionalized with In Situ Green-Synthesized Silver Nanoparticles for Antibacterial Applications
Source: Int J Mol Sci. 2026 Apr 28;27(9):3943. doi: 10.3390/ijms27093943 (PMC13163333; doi:10.3390/ijms27093943)
Supplement: Supplementary file 1 [file ijms-27-03943-s001.zip › ijms-4126913-supplementary.pdf]

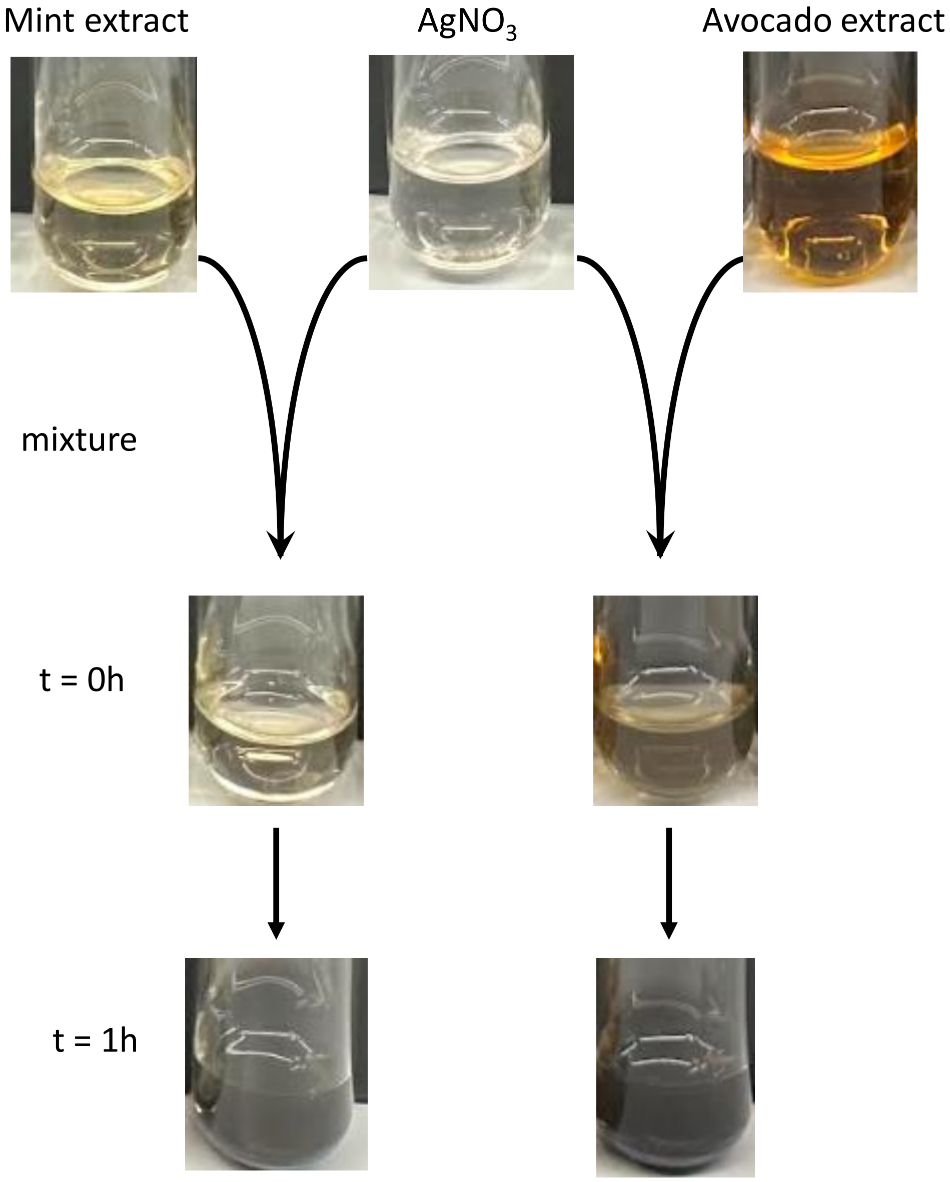

Figure S1. Tubes containing $AgNO_3$ and mint or avocado extracts were mixed. The image shows the color evolution at different times after mixing. After 1 h, a significant color change was observed, consistent with the formation of AgNP.

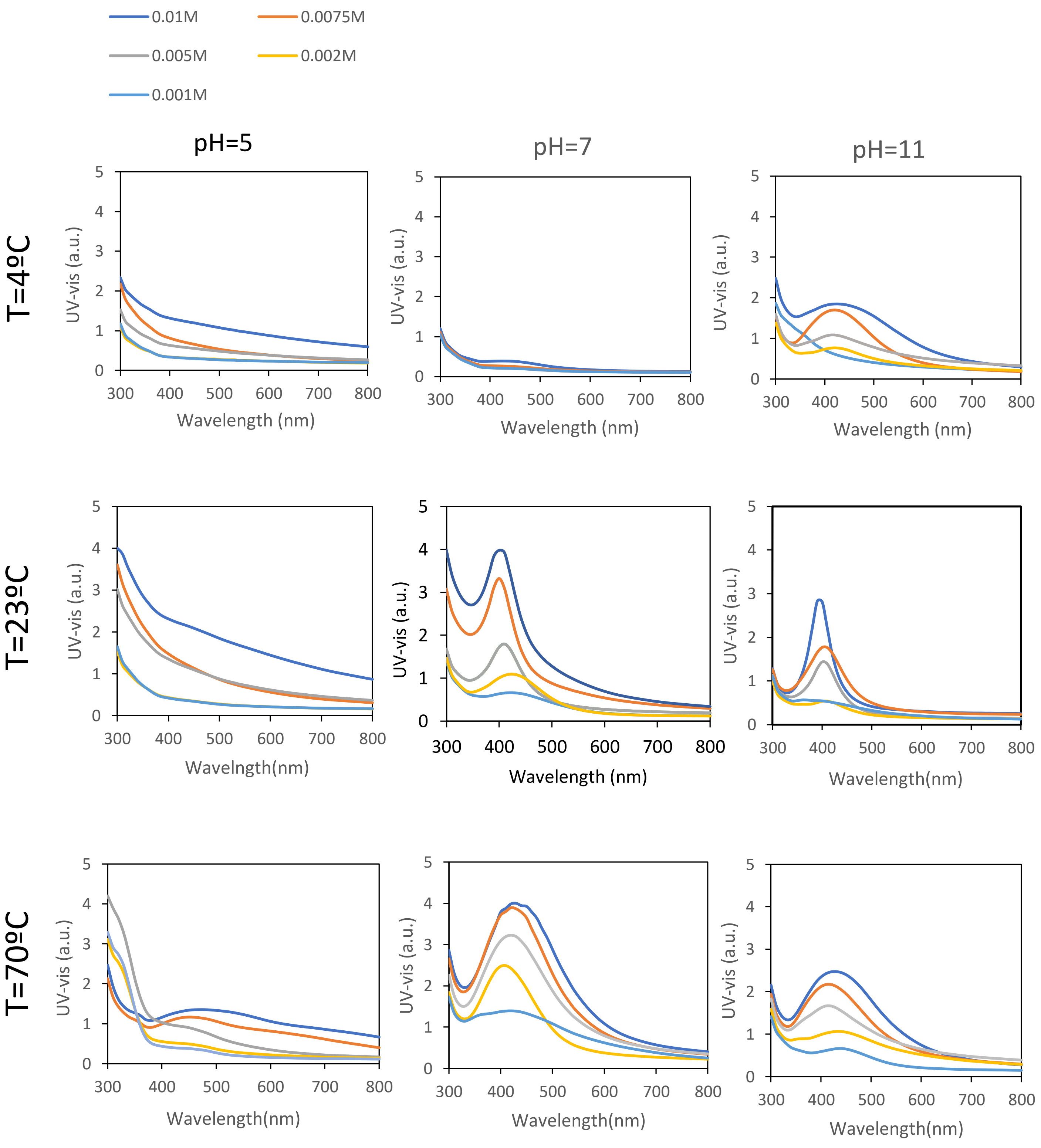

Figure S2. UV–Vis spectra of silver nanoparticle (AgNP) synthesis in aqueous phase obtained by combining different $AgNO_3$ concentrations with the same volume of avocado extract. Syntheses were carried out under various combinations of pH and temperature. The spectra clearly demonstrate the strong influence of these parameters on AgNP synthesis.

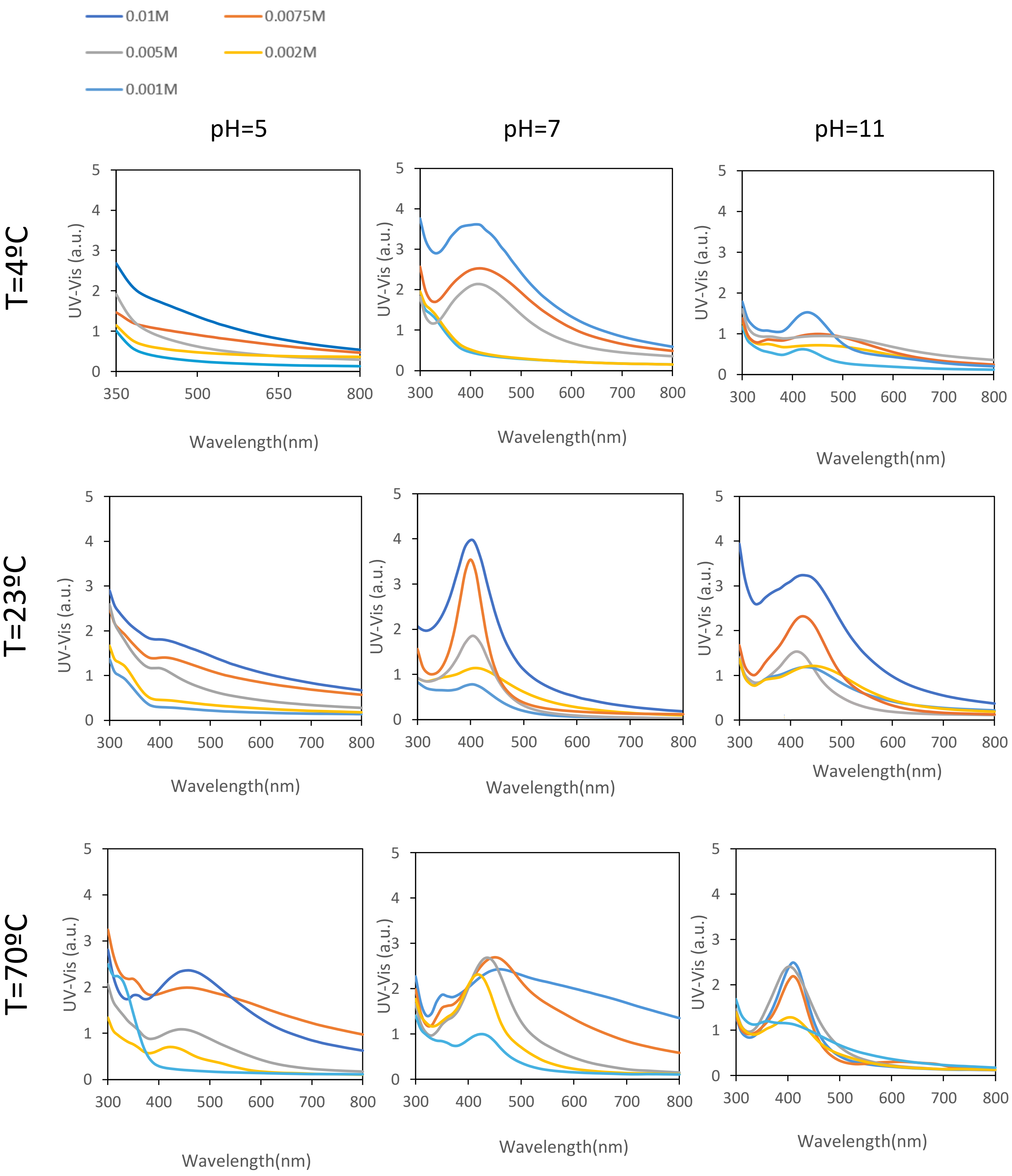

Figure S3. UV–Vis spectra of silver nanoparticle (AgNP) synthesis in aqueous phase obtained by combining different $AgNO_3$ concentrations with the same volume of mint extract. Syntheses were carried out under various combinations of pH and temperature. The spectra clearly demonstrate the strong influence of these parameters on AgNP synthesis.

a

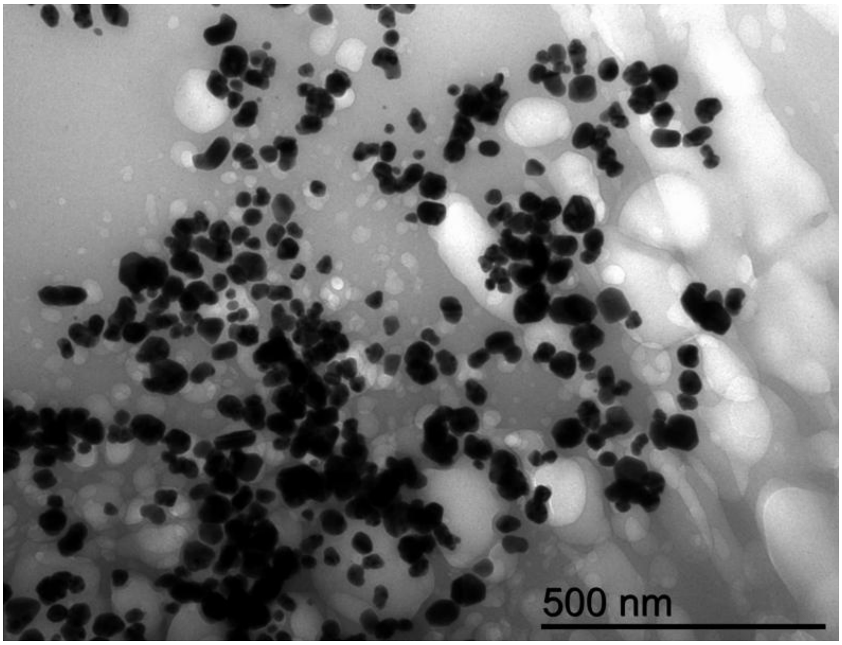

b

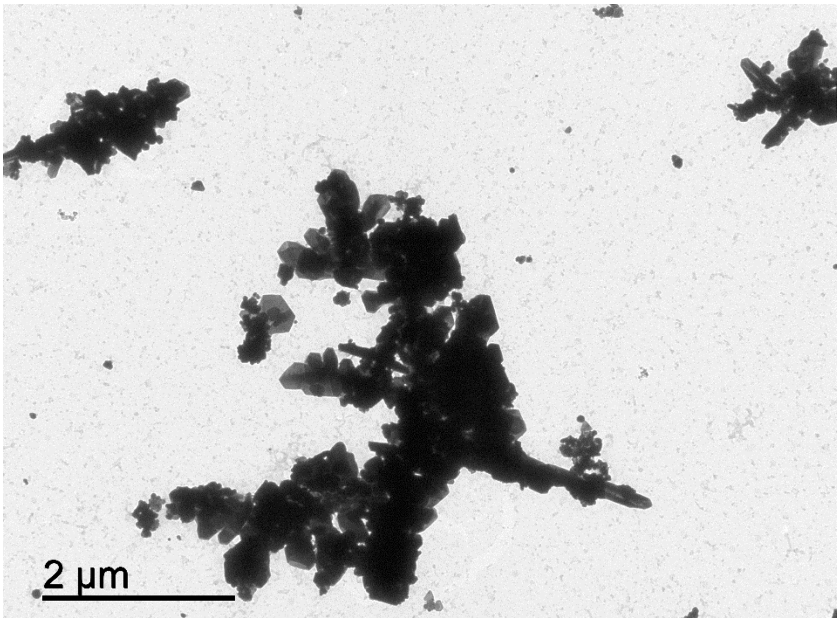

Figure S4. TEM images of silver nanoparticles synthesized in aqueous suspension using plant extracts as reducing agents. **a.** AgNP obtained using avocado seed extract. **b.** AgNP obtained using mint extract.

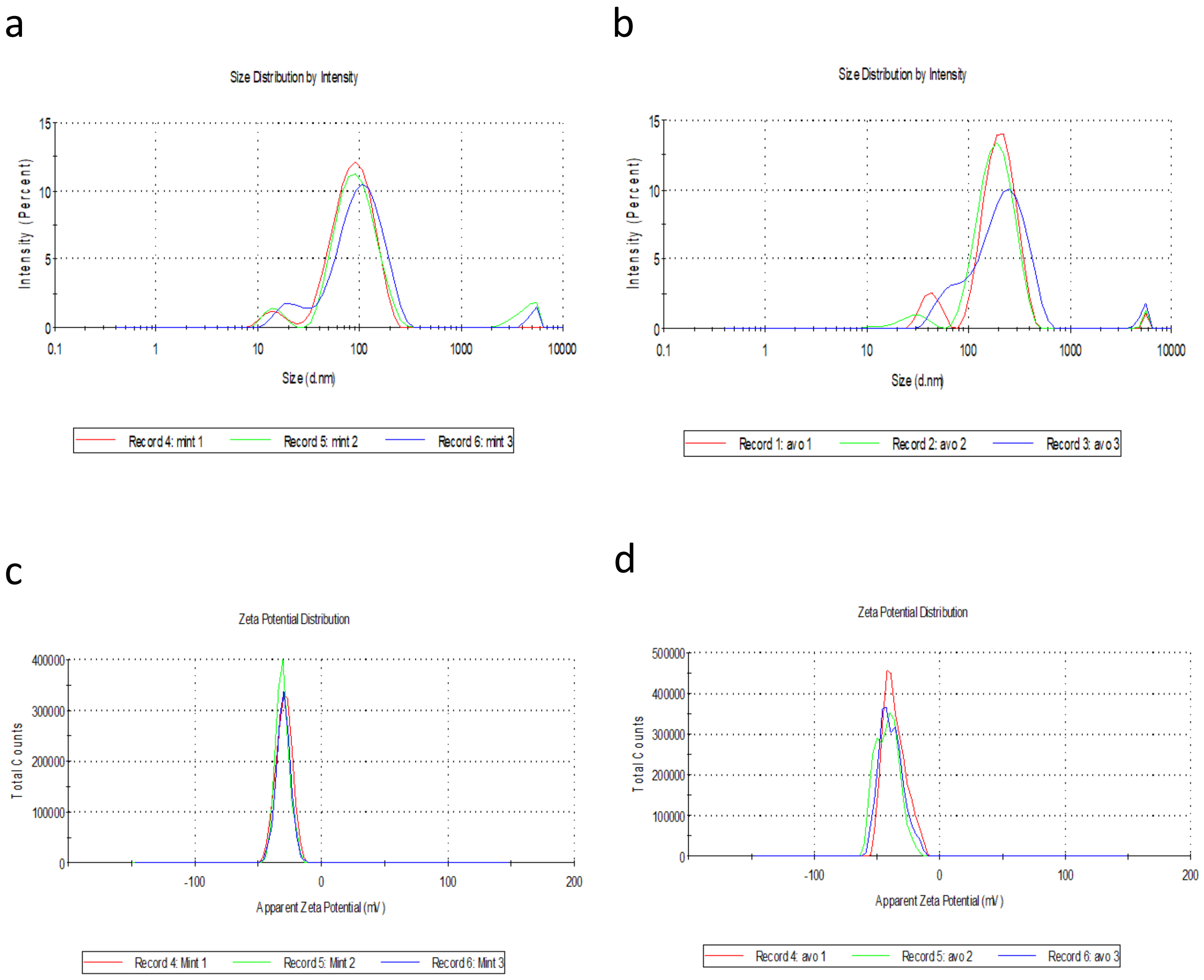

Figure S5. DLS analysis of AgNPs synthesized in an aqueous medium. Hydrodynamic size distribution of AgNPs synthesized with mint extract (a) and avocado seed extract (b). Z-potential values of AgNPs synthesized with mint extract (d) and avocado seed extract (e).

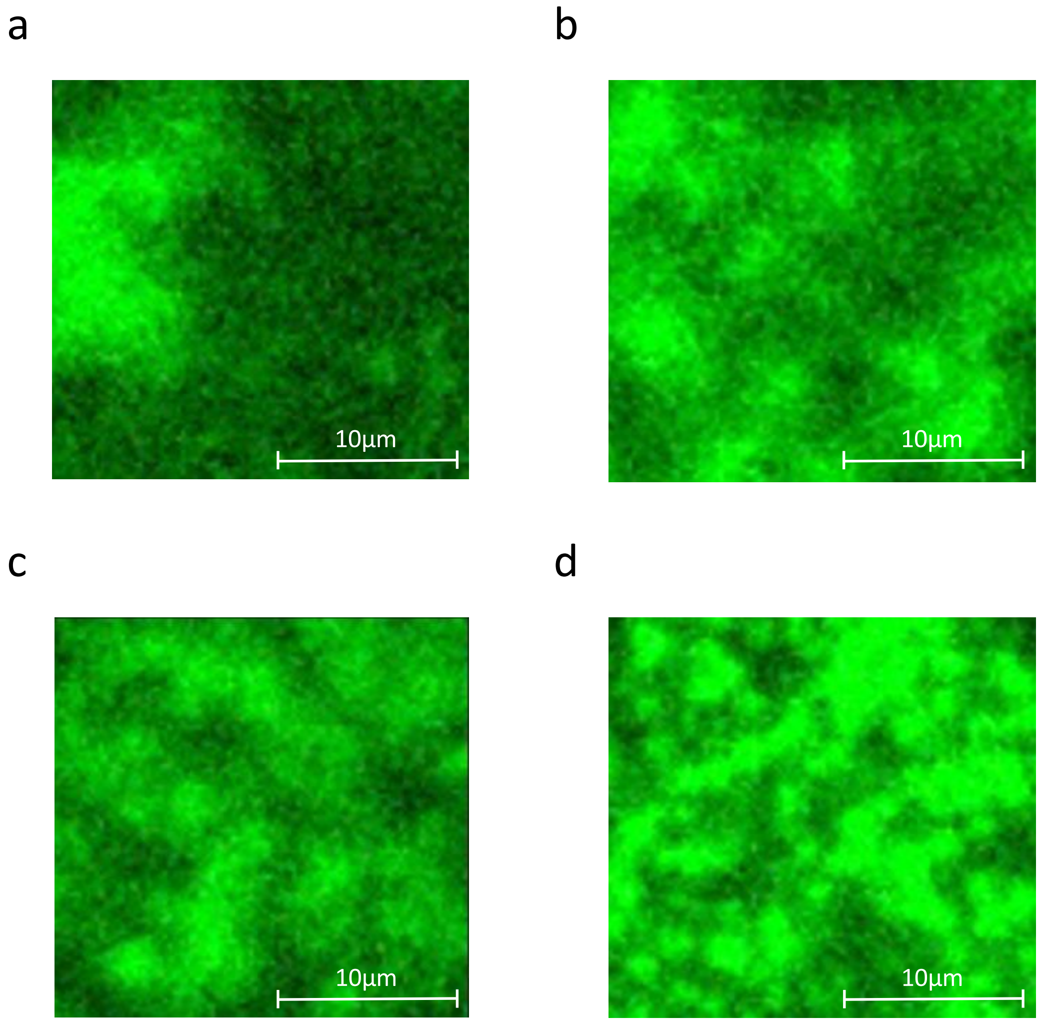

Figure S6. SEM–EDS images of BC membranes. BC membranes loaded by immersion with AgNP synthesized using mint extract (a) and avocado seed extract (b), and BC membranes loaded with AgNP synthesized *in situ* using mint extract (c) and avocado seed extract (d).

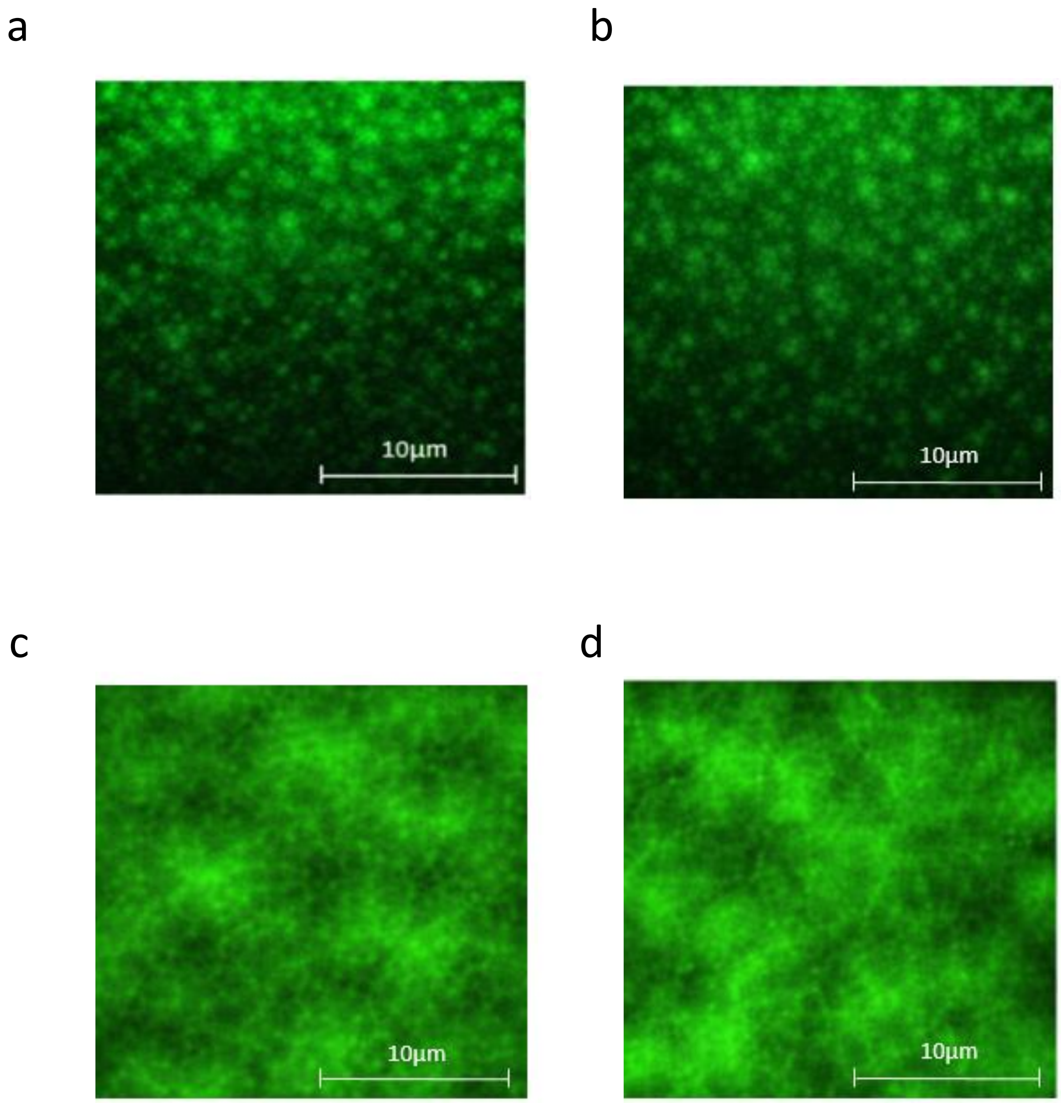

Figure S7. SEM–EDS images of cross-sections of BC membranes. BC membranes loaded by immersion with AgNPs synthesized using mint extract (a) and avocado seed extract (b), and BC membranes loaded with AgNPs synthesized *in situ* using mint extract (c) and avocado seed extract (d).

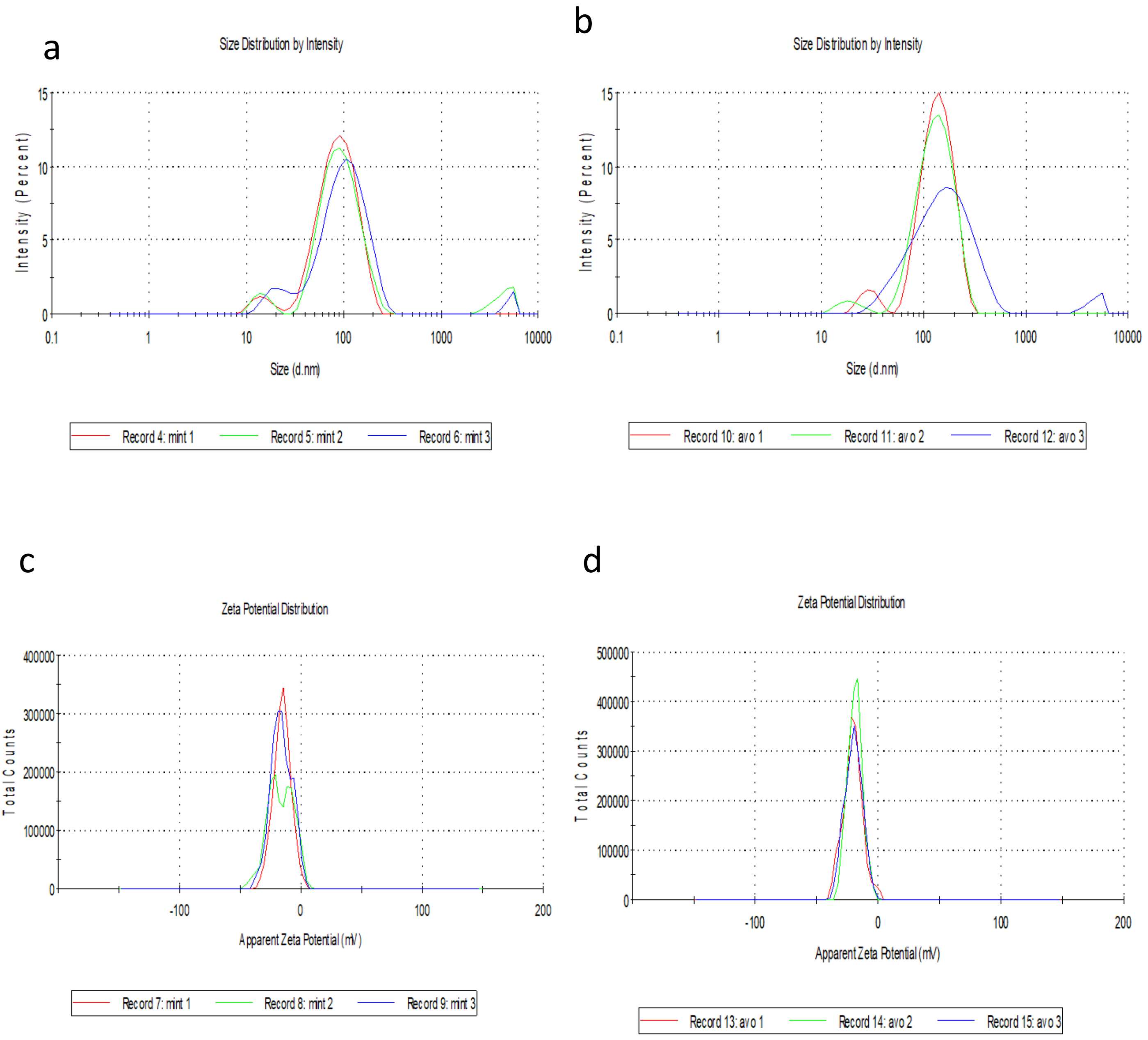

Figure S8. DLS analysis of AgNPs synthesized *in situ* within BC membranes. Hydrodynamic size distribution of AgNPs synthesized using mint extract (a) and avocado seed extract (b). Z-potential values of AgNPs synthesized using mint extract (d) and avocado seed extract (e).

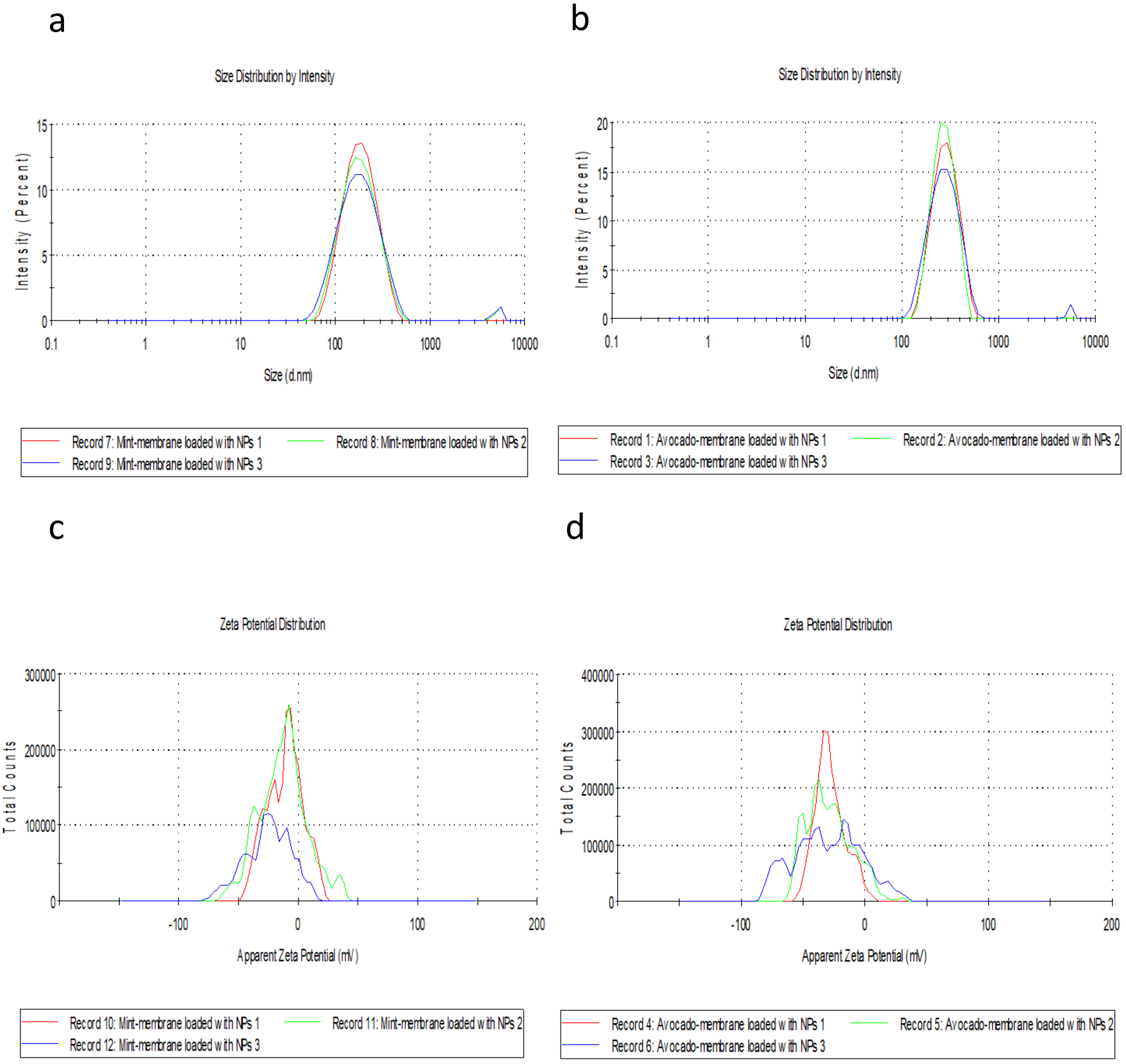

Figure S9. DLS analysis of AgNPs loaded into BC membranes by immersion. Hydrodynamic size distribution of AgNPs synthesized using mint extract (a) and avocado seed extract (b). Z-potential values of AgNPs synthesized using mint extract (d) and avocado seed extract (e).
